# The impact of *Streptococcus thermophilus* IDCC 2201 on gut microbiota and its potential as a prophylactic agent for colorectal cancer

**DOI:** 10.1038/s41598-025-20976-w

**Published:** 2025-10-23

**Authors:** Eoun Ho Nam, Minjee Lee, Hayoung Kim, Donggyu Kim, Yeji Lee, Young Hoon Jung, Jungwoo Yang, Minhye Shin

**Affiliations:** 1 Department of Microbiology, College of Medicine, Inha University, Incheon, 22212 Republic of Korea; 2 Program in Biomedical Science and Engineering, Inha University, Incheon, 22212 Republic of Korea; 3 Ildong Bioscience, Pyeongtaek-si, 17957 Gyeonggi-do Republic of Korea; 4 Food and Bio-industry Research Institute, School of Food Science & Biotechnology, College of Agriculture and Life Sciences, Kyungpook National University, Daegu, 41566 Republic of Korea; 5 Department of Microbiology, College of Medicine, Dongguk University, Gyeongju, 38066 Republic of Korea

**Keywords:** Microbiology, Gastroenterology

## Abstract

Colorectal cancer (CRC) is one of the leading causes of cancer-related deaths. The gut microbiota is known to be closely associated with CRC development, interacting with each other and influencing host metabolism. Probiotic interventions have been applied to prevent CRC; however, the mechanisms underlying their effects on CRC remain unclear. In this study, we evaluated the effects of the probiotic strain *Streptococcus thermophilus* IDCC 2201 on its interactions with gut commensal bacteria and CRC cell viability. *S. thermophilus* inhibited the growth of most bacterial species comprising the human gut microbiota, with the exception of *Bacteroides dorei*. Further studies identified several factors produced by *S. thermophilus*, such as xylan-degrading enzymes and B vitamins, which promoted *B. dorei* growth through nutrient cross-feeding. During co-culture of *S. thermophilus* with individual gut commensals, bioactive compounds such as folate were significantly produced, further inhibiting CRC cell viability along with the culture supernatant of *S. thermophilus*. These findings suggest that *S. thermophilus* IDCC 2201 may serve as a potential prophylactic agent for colon cancer, with its effects mediated through interactions with gut commensal bacteria.

**Supplementary Information:**

The online version contains supplementary material available at 10.1038/s41598-025-20976-w.

## Introduction

Colorectal cancer (CRC) is one of the most common types of cancer worldwide, accounting for approximately 10% of all cancer cases and ranking as the second leading cause of cancer-related deaths^1^. Several factors are known to increase CRC risk, including age, genetic predisposition, and environmental influences^2^. In particular, environmental factors such as pro-carcinogenic dietary metabolites, excessive alcohol consumption, and chronic gut inflammation contribute to tumorigenesis^3^. Recent findings have highlighted the critical role of gut microbiota in CRC development, largely in association with these environmental factors^4,5^.

Residing in close proximity to the colorectal epithelium, gut microbiota interacts with host cells by regulating metabolism and immune responses^6^. *Bacteroides fragilis*, *Fusobacterium nucleatum*, and *Escherichia coli* have been suggested as candidate CRC-associated pathogens^7^. The relative abundance of these bacteria is often elevated in CRC patients, leading to the production of virulence factors such as *B. fragilis* toxin, adhesins FadA/Fap2, and colibactin^8–10^. Notably, these bacteria contribute to disease progression not as individual strains but as groups of microorganisms that collectively exacerbate detrimental effects. Conversely, certain bacteria, primarily probiotics, are depleted in CRC patients and exert protective effects against CRC-associated pathogens.

Probiotic interventions have been explored as potential therapeutics for various health conditions, including pregnancy, inflammation, neuropsychiatric disorders, and cancer^11–13^. The beneficial effects of probiotics are attributed to mechanisms such as immune modulation, stimulation of gut epithelial proliferation, and maintenance of intestinal microbiota homeostasis^14–17^. Specifically, probiotics have been shown to enhance the composition of beneficial bacteria by providing bioactive compounds and to regulate intestinal homeostasis through competitive exclusion of pathogens, thereby influencing host immunity and host-microbe interactions^18^.

Microorganisms form complex communities and interact each other via synergistic, competitive, or antagonistic relationships mediated by nutrient availability, inhibitory compounds, and signaling molecules^19^. For example, Bacteroidales degrade plant polysaccharides into monomers that support the growth of other species, often deriving reciprocal benefits in the process^20^. Additionally, some bacteria produce intermediate metabolites, such as short-chain fatty acids (SCFAs), which can be further utilized by other microorganisms^21^. Among the various nutrient sources facilitating bacterial interactions, vitamins play a crucial role as key intermediates. In silico predictions indicate that approximately 20% of a typical microbial population is auxotrophic for each B vitamin, while experiments have confirmed that prototrophic bacteria can support auxotrophs under nutrient-deficient conditions^22,23^.

Recent studies on the anticancer effects of *Streptococcus thermophilus* have been steadily increasing^24–27^. Li et al. found that β-galactosidase secreted by *S. thermophilus* inhibited tumor progression by modulating oxidative phosphorylation and Hippo pathway kinases^24^. Additionally, *S. thermophilus* strains isolated from dairy environments have demonstrated anticancer activity, possibly linked to folate production^25^. In mouse fibrosarcoma and neuroblastoma cells, *S. thermophilus* exhibited antitumor effects through the activation of T-lymphocytes^26,27^. However, the precise mechanisms underlying these effects remain poorly understood.

In this study, we evaluated the effects of *S. thermophilus* IDCC 2201 on the growth of individual gut commensal bacteria and investigated its intra-species-specific interactions. Specifically, we elucidated the growth-promoting mechanism of *S. thermophilus* on *Bacteroides dorei*. Finally, we assessed its anticancer effects via the production of potential bioactive metabolites, suggesting *S. thermophilus* as a promising prophylactic agent for CRC prevention.

## Methods

### Bacterial strains and culture conditions

The bacterial strains used in this study are listed in Table S1. *S. thermophilus* IDCC 2201 was obtained from Ildong Biosciences, while *S. thermophilus* type strains of KCTC 21173 (ATCC 19258) and KCTC 3779 (ATCC BAA-250) were purchased from the Biological Resource Center (Korean Collection for Type Cultures KCTC, Jeongeup-si, South Korea), and grown in modified GMM at 37 ℃ under anaerobic conditions for 24 h in a static incubator^28^. For a synthetic human gut bacterial community, 17 species of the most common bacteria present in the human gut were obtained from KCTC (Table S1) and maintained in strain-specific media under anaerobic conditions. Most strains were grown in Tryptic soy broth hemin menadione supplemented with 0.05% (w/v) L-cysteine and 5% (v/v) commercial sheep blood (MBcell, Seoul, South Korea), except for *Bifidobacterium longum subsp.infantis*,* Lacticaseibacillus rhamnosus*,* Lacticaseibacillus casei*,* Streptococcus thermophilus*,* Bacteroides ovatus*,* Parabacteroides distasonis*, and *Akkermansia muciniphila. B. infantis* was grown in BL broth (BD Difco, Sparks, MD, USA), while *L. rhamnosus*,* L. casei*, and *S. thermophilus* were cultured in MRS broth (BD Difco). Other strains were cultivated in commercial chopped meat broth media supplemented with hemin, menadione, and vitamin K1 (MBcell).

### GMrepo analysis

Metagenome data from fecal samples of patients with colorectal neoplasms and healthy controls were retrieved from the GMrepo database (https://gmrepo.humangut.info). The data were obtained using “Colorectal Neoplasms” as the keyword, without considering patient age, BMI, gender, or nationality. All microbiome data retrieved from the GMrepo database were confirmed as curated projects, and the confounders were evaluated based on Cox regression model. The odds ratios (95% CI) of confounders, including age, sex, and BMI, were 1.046 (1.035 to 1.058), 0.8654 (0.6666 to 1.118), and 1.016 (0.9785 to 1.053), respectively.

### Co-culture experiments

A single colony of each strain was grown to saturation in its strain-specific medium for 72 h and was then washed three times with 0.85% (w/v) NaCl to remove impurities. The cells were diluted to an OD_600_ of 0.1 in 5 ml of fresh modified SHIME medium (mSHIME). Pairwise co-cultures were established by pooling the diluted cultures in a 1:1 ratio. For mono-cultures, only a single bacterial culture was used. The mSHIME medium comprised 1.2 g/l arabinogalactan, 2 g/l pectin, 0.5 g/l xylan, 0.4 g/l glucose, 3 g/l yeast extract, 1 g/l peptone, 2 g/l mucin, 0.5 g/l cysteine-HCl, 4 g/l starch, 1 ml/l Tween 80, 1 ml/l Microelement solution, and 1 ml/l Vitamin solution. The Microelement solution contained 500 mg/l MnSO_4_, 100 mg/l FeSO_4_, 100 mg/l CoSO_4_, 100 mg/l ZnSO_4_, 10 mg/l CuSO_4_, 10 mg/l Alk(SO_4_), 10 mg/l H_3_BO_3_, 100 mg/l Na_2_MoO_4_, 100 mg/l NiCl_2_, and 10 mg/l Na_2_SeO_3_. Vitamin solution included 1 mg/l menadione, 2 mg/l biotin, 10 mg/l pantothenate, 5 mg/l nicotinic acid, 0.5 mg/l vitamin B_12_, 4 mg/l thiamine, and 5 mg/l folic acid. The cells were grown in an anaerobic chamber with 10% CO_2_, 5% H_2_, and 85% N_2_. Growth was monitored by measuring OD_600_ and CFU counting. The bacterial interaction was determined by calculating the ratio of CFU for individual strain in co-culture with *S. thermophilus* relative to monoculture^29^. To distinguish the two bacterial species after co-culture, the growth ability of each strain on different culture media was testes, as shown in Table S2. For CFU counting of *S. thermophilus*, BS or TOS-MUP media were used. After 72-hour growth, the strain-specific CFUs from co-culture divided by the value from mono-culture was calculated as the abundance ratio, r_bm_ (r_i_,_bm=_ $\:\frac{CFUi(co,\:72h)}{CFUi(mono,72h)}$).

### Co-culture of bacterial community experiments

A single colony of each strain was grown to saturation in its strain-specific medium for 72 h and was then washed three times with 0.85% (w/v) NaCl to remove impurities. The cells were diluted to an OD_600_ of 0.1 in fresh modified SHIME medium (mSHIME). Co-cultures of the bacterial community were established by mixing 50 µl of each bacterial culture in a final 5 ml of culture volume. For the control culture, only the 17 bacterial strains were included, while *S. thermophilus* was added in the co-culture. The cells were grown in the mSHIME medium anaerobically with 10% CO_2_, 5% H_2_, and 85% N_2_ at 37 °C for 72 h.

Growth was measured based on the 16S rRNA sequence analysis. Genomic DNA was extracted from the cell pelles using DNeasy PowerSoil Pro Kit (Qiagen, Hilden, Germany) according to the manufacturer’s instructions. DNA was quantified using Quant-IT^TM^ PicoGreen^®^ dsDNA Assay Kit (Thermo Fisher Scientific, Waltham, MA, USA). The sequencing libraries were prepared according to the PacBio Sequel II3 sequencing Library protocols (Menlo Park, CA, USA) to amplify the 27 F and 1492R region. The universal primer pair with asymmetric barcoded adpaters sequences were as follows: 27F-F: 5’- AGRGTTYGATYMTGGCTCAG − 3’ (16S Amplicon PCR Forward Primer) and 5’- RGYTACCTTGTTACGACTT − 3’ (16S Amplicon PCR Reverse Primer).

The sequences were analyzed using Sequen II Sequencing kit 2.0 and SMRT cells 8 M Tray, and Cutadapt (v3.2), DADA2 (v1.18.0) package of the R (v4.0.3) (https://www.r-project.org/) program were used for data preprocessing. For comparative analysis of microbial communities, read counts were normalized by applying subsampling based on the read count of the sample with the minimum read count among all samples using QIIME (v1.9) program.

### Dinitrosalicylic acid (DNS) assay

The DNS assay was conducted according to previously described procedures with slight modifications^30^. Extracellular and intracellular fractions were prepared from culture supernatant and crude extract of cell pellets of *S. thermophilus*, respectively. Samples were incubated with substrates including arabinogalactan, pectin, xylan, and mucin. After incubation, 500 µl of DNS reagent was added, and samples were heated for 5 min at 90 ℃. Samples were cooled down to room temperature, diluted to an appropriate absorbance range, and absorbance was recorded at 575 nm using a microplate reader.

### Metabolome analysis

Probiotic culture supernatant (750 µl) was diluted in 2.25 ml of ice-cold methanol and vortexed for 1 min, followed by centrifugation at 13,000 g for 10 min at 4 °C. One hundred microliters of supernatant was collected, concentrated to dryness in a vacuum concentrator, and stored at −80 °C until required. Samples were derivatized by adding 30 µl of a solution of 20 mg/ml methoxyamine hydrochloride in pyridine (Sigma, St. Louis, MO, USA) for 90 min at 30 °C, and then adding 50 µl of N, O-bis(trimethylsilyl)trifluoroacetamide (BSTFA; Sigma) and heating for 30 min at 60 °C. A mixture of alkane standards and fluoranthene was used as retention indices and an internal standard, respectively. GC-MS analysis was conducted using a Thermo Trace 1310 GC (Thermo, Waltham, MA, USA) coupled to a Thermo ISQ LT single quadrupole mass spectrometer (Thermo). GC was performed using a DB-5MS column (60-m length, 0.25 mm i.d., and 0.25-µm film thickness) (Agilent, Santa Clara, CA, USA). Derivatized samples were injected at 300 °C using a split ratio of 1:5, and metabolites were separated using a helium flow of 1.5 ml using the following oven program; 2 min at 50 °C, 50 °C to 180 °C at 5 °C/min, 8 min at 180 °C, 180 °C to 210 °C at 2.5 °C/min, 210 °C to 325 °C at 5 °C/min, and 10 min at 325 °C. Mass spectra were acquired in the scan range 35–650 m/z at 5 spectra per sec in electron impact ionization mode and an ion source temperature of 275 °C. Spectra were processed using Thermo Xcalibur and AMDIS softwares with automated peak detection, and metabolites were identified by matching mass spectra and retention indices using the NIST Mass spectral search program (version 2.0, Gaithersburg, MD, USA) and MS-DIAL (http://prime.psc.riken.jp/compms/msdial/main.html). Relative metabolite intensities were normalized by the sum of identified peaks.

### Quantification of B vitamins

The culture supernatant was concentrated using a vacuum concentrator (Vision VS-802, VISIONBIONEX, Bucheon-si, South Korea) for 24 h at room temperature. The concentrated samples were re-dissolved in mobile phase A, concentrated ten-fold, and then filtered through a 0.2 μm syringe filter prior to HPLC analysis (SHISEIDO Nanospace Sl-2, SHISEIDO, Osaka, Japan). For the analysis and quantification of vitamins, a CapCellPAK 120UG C18 column (Osaka Soda, Osaka, Japan) was used. Mobile phase A was prepared by adding 10 ml of PIC (paired-ion chromatography) reagent to 500 ml of ultrapure water, and then filtering it through a 0.2 μm syringe filter after degassing. Mobile phase B was prepared by adding 10 ml of PIC reagent to 500 ml of 60% methanol and filtering through a 0.2 μm syringe filter after degassing. The PIC reagent was formulated by dissolving 1 g of 1-heptanesulfonic acid (Sigma) in a solution of 10 ml of distilled water and 10 ml of acetic acid (Sigma). The flow rate was set at 0.5 ml/min with the following gradient elution: 0–3 min, 5% B; 3–4 min, 13% B; 4–12 min, 20% B; 12–15 min, 25% B; 15–17 min, 30% B; and 17–20 min, 33% B; 20–24 min, 40% B; 24–30 min, 45% B; 30–32 min, 45% B; 32–35 min, 40% B; 35–37 min, 40% B; 37–45 min, 60% B; 45–50 min, 100% B; 50–55 min, 5% B. The column temperature was consistently held at 40 °C. For each analysis, 20 µl of the sample was injected, and peak detection occurred at 270 nm using a UV detector. It is noted that B vitamins have varying detection range depending on the specific vitamin, typically between 210 and 360 nm. It is known that wavelengths between 265 and 280 nm are favorable for their simultaneous detection. In line with these findings, 270 nm has been widely adapted in many studies, offering relatively good sensitivity^31,32^.

### Quantification of SCFAs

The culture supernatant was obtained by centrifugation at 6,000 rpm and 4 ℃ and was then concentrated twice using a vacuum concentrator (Eppendorf Concentrator plus™, Eppendorf, Hamburg, Germany). The sample was filtered through a 0.45 μm syringe filter and subsequently analyzed using high-performance liquid chromatography (HPLC; Agilent 1260, Agilent Technologies, Santa Clara, CA, USA). For the separation and quantification of SCFA, an Aminex HPX-87 H column (300 mm x 7.8 mm, 9 μm particle size; Bio-Rad Laboratories, Hercules, CA, USA) was used. The mobile phase consisted of 0.005 N sulfuric acid with a flow rate of 0.6 ml/min. The column temperature was maintained at 60 ℃, and a10 µl sample was injected. Peaks were detected using both a UV detector and a refractive index detector (RID) set at 210 nm.

### Cell viability assay

Human colon adenocarcinoma cell lines HCT116 and HT-29 were obtained from the American Type Culture Collection (ATCC, Manassas, VA, USA) and the Korean Cell Line Bank (KCLB, Seoul, South Korea), respectively. The cell lines were cultured in Dulbecco’s modified Eagle’s medium (DMEM, GenDEPOT, Barker, TX, USA) and RPMI1640 medium (GenDEPOT), respectively, supplemented with 10% (v/v) fetal bovine serum (GenDEPOT) and 1% penicillin/streptomycin in a 5% CO_2_ incubator.

The cell viability assay was based on MTT, 3-(4,5-dimethylthiazol-2-yl)−2,5-diphenyltetrazolium bromide, following the product manual with slight modifications. Cells (2 × 10^3^) in each well of a 96-well plate were treated with culture supernatants at different concentrations (5%, 10%, 20%) for 48 h. Subsequently, 10 µl of MTT solution was added and further incubated for 4 h in a 5% CO_2_. The medium was then removed, and the formazan crystals formed were dissolved in 200 µl of dimethyl sulfoxide (DMSO, Sigma). Finally, cell viability was determined by measuring absorbance at 570 nm using an ELISA Reader.

### Statistical methods

Experimental data from all studies were evaluated by unpaired Student’s t-test and One-way ANOVA using GraphPad Prism 9 (San Diego, CA, USA). Differences were considered significant when *p* or P values < 0.05 (*) or < 0.01 (**). Multivariate analysis was performed using MetaboAnalyst 5.0 (https://www.metaboanalyst.ca/)^33^. Heatmaps were generated using the R heatmap package (https://github.com/raivokolde/pheatmap).

## Results

### *S. thermophilus* is negatively associated with CRC

**Fig. 1:**
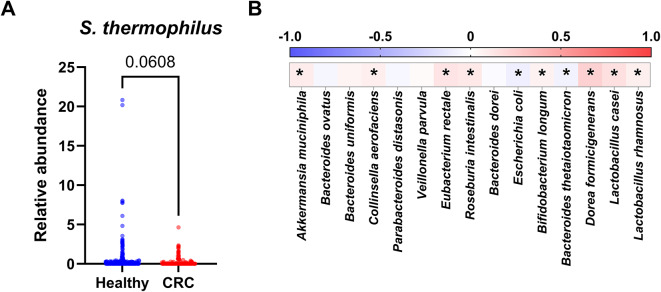
Depletion of *S. thermophilus* in the stool microbiome of patients with colorectal neoplasm. (**A**) Relative abundance of *S. thermophilus* in healthy control and patients with colorectal neoplasm (CRC) based on GMrepo database. Difference between healthy controls and CRC patients are indicated by p-value using Mann-Whitney U test. (**B**) Spearman correlation analysis of relative abundance between *S. thermophilus* and individual bacterial species present as commensals in human intestine. Data were derived from GMrepo database. Colors range from blue (negative correlation) to red (positive correlation). Significant correlations are marked by an asterisk when *P* < 0.05.

Previous studies have suggested that *S. thermophilus* reduces the risk of CRC^24,34^. To further investigate the potent relationship between *S. thermophilus* and CRC, we first compared the relative abundance of stool bacterial species in patients with colorectal neoplasms and healthy controls using publicly available databases. The data were retrieved from the GMrepo database, which contains curated and consistently annotated human gut metagenomes^35^. A total of 341 samples obtained by whole-genome metagenomics sequencing (40 patients with colorectal neoplasm and 301 healthy controls). The relative abundance of *S. thermophilus* was lower in the patient group compared to the control group (Fig. 1A).

To identify potential associations between *S. thermophilus* and bacterial species within the human gut microbiota, we selected 17 bacterial species that are relatively abundant according to existing datasets^36–39^ (Fig. S1). These strains belong to various phyla, including Actinomycetota, Pseudomonadota, Bacillota, Verrucomicrobiota, and Bacteroidota. A comparison of their relative abundances in human stool samples revealed that *S. thermophilus* exhibited weak but significant positive correlations with *Dorea formicigenerans*, *Lacticaseibacillus casei*, and *Eubacterium rectale*, while showing a negative correlation with *Escherichia coli* and *Bacteroides thetaiotaomicron* (Fig. 1B). These results suggest that *S. thermophilus* is depleted in patients with colorectal neoplasms and that its presence is associated with specific bacterial species in the human gut microbiota.

### Most gut microbial species compete with *S. thermophilus*, but *B. dorei* exhibits a commensal relationship

Microorganisms within a community interact through competition or nutrient sharing, influencing overall microbial function and host health^40^. To assess the effects of *S. thermophilus* on bacterial interactions, we conducted pairwise co-cultures over 72 h and analyzed competition between strains by calculating the individual abundance ratio (r_bm_) in co-culture versus mono-culture^29^. It is noted that growth rates and entry into the stationary phase varied among strains when cultured in media simulating the human gut environment; however, most strains reached the stationary phase within 72 h (Fig. S1C).

**Fig. 2:**
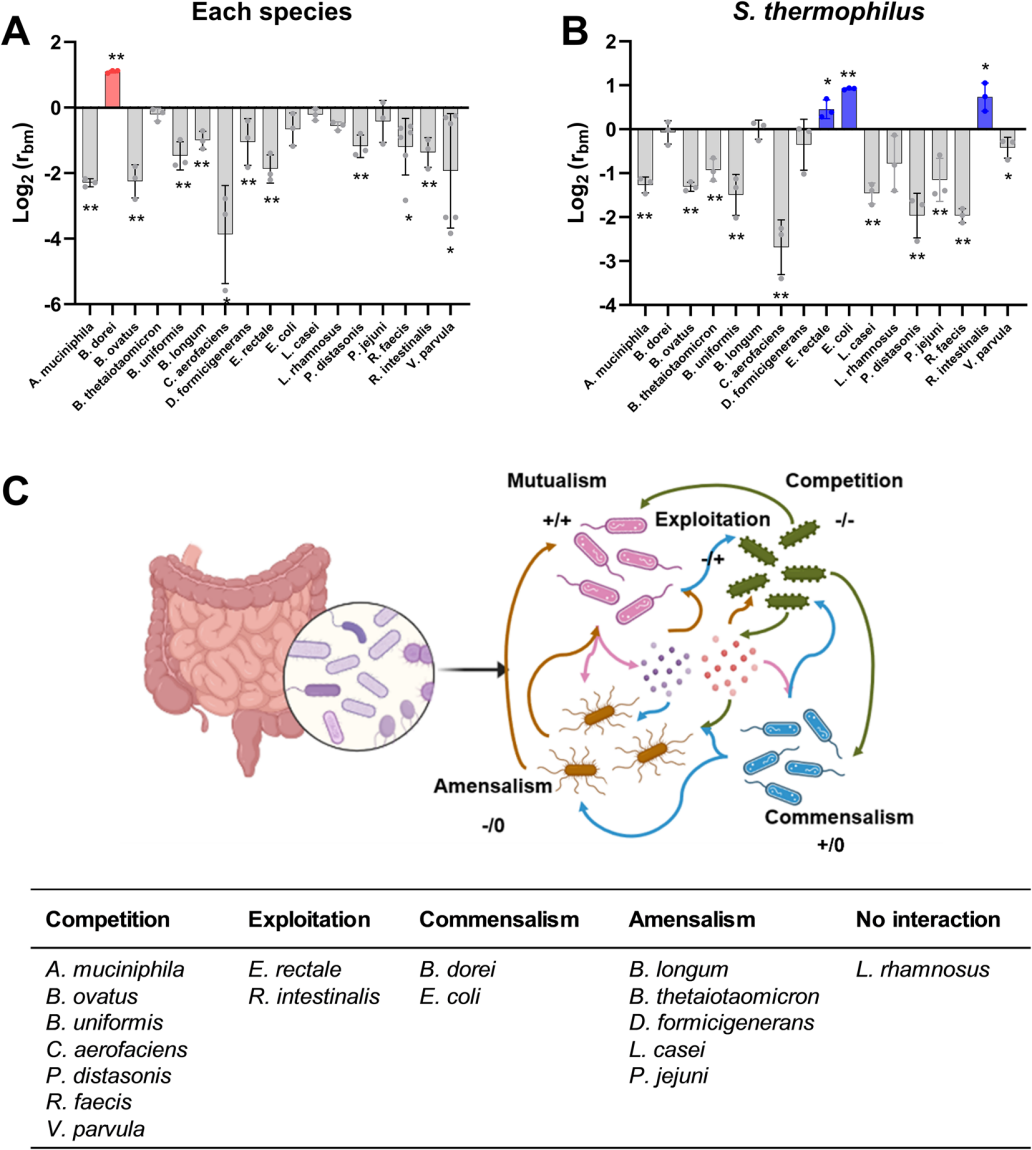
Pairwise interactions between *S. thermophilus* and individual bacterial strains composing human gut microbial community. (A and B) Individual abundance ratio (r_bm_) of CFU in co-culture compared to its mono-culture for each strain (**A**) and *S. thermophilus* (**B**). Each strain and *S. thermophilus* were cultured in a 1:1 ratio for 72 h, and numbers of CFU were determined. The r_bm_ = 1 means no change, and r_bm_ > 1 and r_bm_ < 1 mean an increase and decrease in CFUs in the co-culture compared to the monoculture, respectively. Significant differences between mono- and co-culture are indicated by asterisks at the 95% (*) and 99% (**) significance levels using Student’s t-test. (**C**) Summary of microbial interactions between each strain and *S. thermophilus* based on the r_bm_ results.

Strains interacting with *S. thermophilus* were categorized as having positive (r_bm_>1), negative (r_bm_<1), or neutral (no significant difference) interactions. Most strains exhibited a negative interaction with *S. thermophilus* (Fig. 2A). Notably, *Bacteroides dorei* showed a positive interaction with *S. thermophilus*, yet *S. thermophilus* did not have a growth benefit from *B. dorei*. Interestingly, *S. thermophilus* exhibited enhanced growth when co-cultured with *E. coli*, *E. rectale* and *Roseburia intestinalis* (Fig. 2B).

Microbial interactions can be classified into five categories: competition (-/-), where both species experience reduced growth; amensalism (-/0), where one species is inhibited while the other remains unaffected; exploitation (-/+), where one species benefits at the expense of the other; commensalism (+/0), where one species benefits without affecting the other; and mutualism (+/+), where both species benefit^19^. Among the 17 bacterial species analyzed, most exhibited either competitive or amensal interactions with *S. thermophilus* (Fig. 2C). Given that cross-feeding interactions can be commensal or mutualistic, *B. dorei* exhibited a commensal relationship with *S. thermophilus*, while *E. coli* conferred a growth advantage to *S. thermophilus*.

In order to test whether this phenomenon is also observed in the microbial community, we conducted analysis of microbial changes among the 17 most common bacterial species present in the human gut after treatment with *S. thermophilus*. All strains were added to the community at the same cell number, and their presence was assessed using next-generation sequencing based on 16S rRNA genes. As shown in Fig. S2, several bacterial strains, including *B. dorei* and *D. formicigenerans*, showed increased growth in the community upon addition of *S. thermophilus*, whereas the growth of *L. rhamnosus* was reduced. It is noted that only 7 strains were detectable after in vitro cultivation of the 17 strains. Considering that the growth of *B. dorei* also increased in a pairwise co-culture with *S. thermophilus*, its growth stimulation in the microbial community was confirmed. In summary, most gut bacterial species competed with *S. thermophilus*, but *B. dorei* exhibited a commensal relationship.

### Xylan-degrading activity in the culture supernatant of *S. thermophilus* promotes the growth of *B. dorei*

**Fig. 3:**
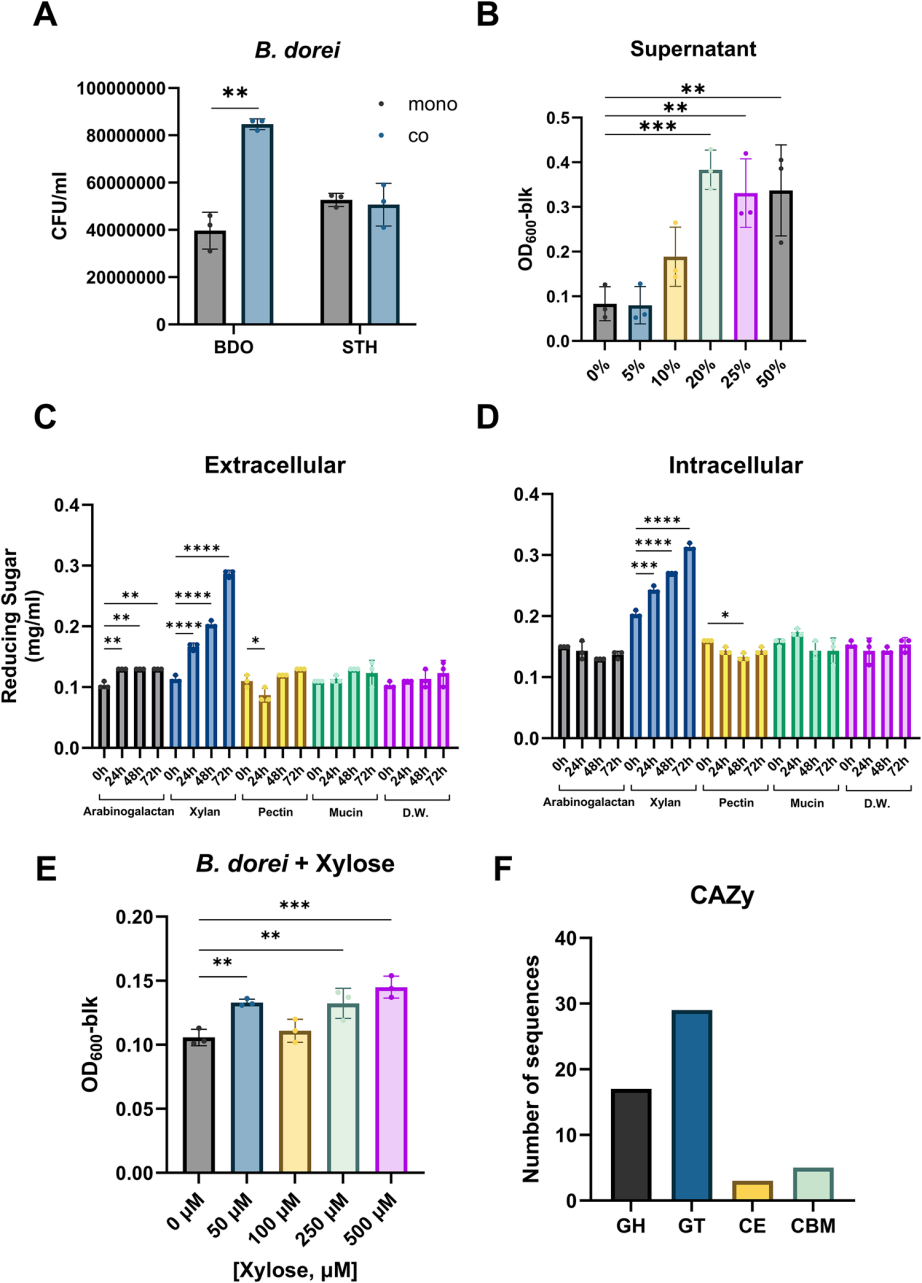
Promotion of *B. dorei* growth by xylan-degrading activity of *S. thermophilus*. (**A**) Growth of *B. dorei* and *S. thermophilus* in mono- and co-culture. Significant differences between mono- and co-culture are indicated by asterisks at the 95% (*) and 99% (**) significance levels using Student’s t-test. (**B**) Growth of *B. dorei* with treatment of the culture supernatant of *S. thermophilus*. Significant differences are indicated by asterisks at the 95% (*), 99% (**), and 99.9% (***) significance levels using one-way ANOVA with Dunnett’s post-hoc analysis. (C and D) Production of reducing sugars from carbohydrates by extracellular (**C**) and intracellular (**D**) fractions of *S. thermophilus*. Significant differences are indicated by asterisks at the 95% (*), 99% (**), 99.9% (***), and 99.99% (****) significance levels using one-way ANOVA with Dunnett’s post-hoc analysis for each carbohydrate source. (**E**) Growth of *B. dorei* with treatment of the xylose. Significant differences are indicated by asterisks at the 99% (**) and 99.9% (***) significance levels using one-way ANOVA with Dunnett’s post-hoc analysis. (**F**) Number of carbohydrate-active enzymes in the *S. thermophilus* IDCC 2201 genome. GH, glycoside hydrolase; GT, glycosyltransferase; CE, carbohydrate esterase; and CBM, carbohydrates-binding module.

*B. dorei* is a commensal gut bacterium that contributes to gut health by alleviating colitis, suppressing proinflammatory immune responses, and aiding in complex carbohydrate digestion^41–43^. As shown in Fig. 3A, co-culture with *S. thermophilus* promoted the growth of *B. dorei* without affecting the growth of *S. thermophilus*. To identify the molecular mechanism by which *S. thermophilus* promotes *B. dorei* growth, we tested the effect of *S. thermophilus* culture supernatant on *B. dorei* (Fig. 3B). The bacterial growth significantly increased with the addition of supernatant in a dose-dependent manner, implicating that specific molecules in the supernatant enhance *B. dorei* growth. In order to test if this result is unique to *S. thermophilus* IDCC 2201, we compared the effects of *S. thermophilus* on the growth of *B. dorei* across different strains, including *S. thermophilus* KCTC 21173 and KCTC 3779. As shown in Fig. S3A, the *S. thermophilus* type strains also influenced the growth of *B. dorei* in a similar manner to IDCC 2201, although the effect was most pronounced with IDCC 2201.

A recent study by Li et al. reported that *S. thermophilus* inhibits colorectal tumorigenesis by secreting β-galactosidase, which provides lactose and subsequently promotes probiotic abundance^24^. To assess whether *S. thermophilus* supplies fermentable sugars by degrading carbohydrate polymers, we quantified reducing sugars liberated by carbohydrate-hydrolyzing enzymes. The mSHIME medium, designed to mimic the gastrointestinal environment, contains complex carbohydrates, proteins, mucins, minerals, and vitamins^44^. We tested arabinogalactan, xylan, and pectin as potential substrates, along with mucin as a possible source of carbohydrates when degraded. Streptococcal crude enzymes were prepared as extracellular and intracellular fractions. Among the tested carbohydrates, only xylan was degraded by the crude enzymes in both fractions (Fig. 3C and D). Additionally, growth of *B. dorei* increased dose-dependently with xylose supplementation, suggesting that xylose liberated by *S. thermophilus* xylan-degrading enzymes promotes the growth (Fig. 3E). Analysis of the CAZy database revealed that *S. thermophilus* IDCC 2201 may harbor a glucosyl hydrolase family GH1 enzyme, potentially a xylan exo-β−1,4-xylosidase (EC 3.2.1.17) (Fig. 3F). Collectively, these results suggest that xylan-degrading enzymes in the culture supernatant of *S. thermophilus* contribute to promotion of *B. dorei* growth.

### Metabolites in the culture supernatant of *S. thermophilus* affect the growth of *B. dorei*

In cooperative microbial interactions, the sharing of metabolites plays a crucial role in species fitness and the shaping of gut microbial communities^19^. In addition to secretory enzymes, we hypothesized that metabolites produced by *S. thermophilus* could be shared with *B. dorei*, boosting its growth. To test this hypothesis, we first analyzed small molecules present in the culture supernatant using GC-MS and identified metabolites that increased in abundance compared to the blank medium. A total 134 metabolites were identified, including organic acids, amino acids, free fatty acids, and nucleotides (Fig. S4). Notably, sugars were not included among the identified metabolites due to the limitations of GC-MS in distinguishing stereoisomers.

**Fig. 4:**
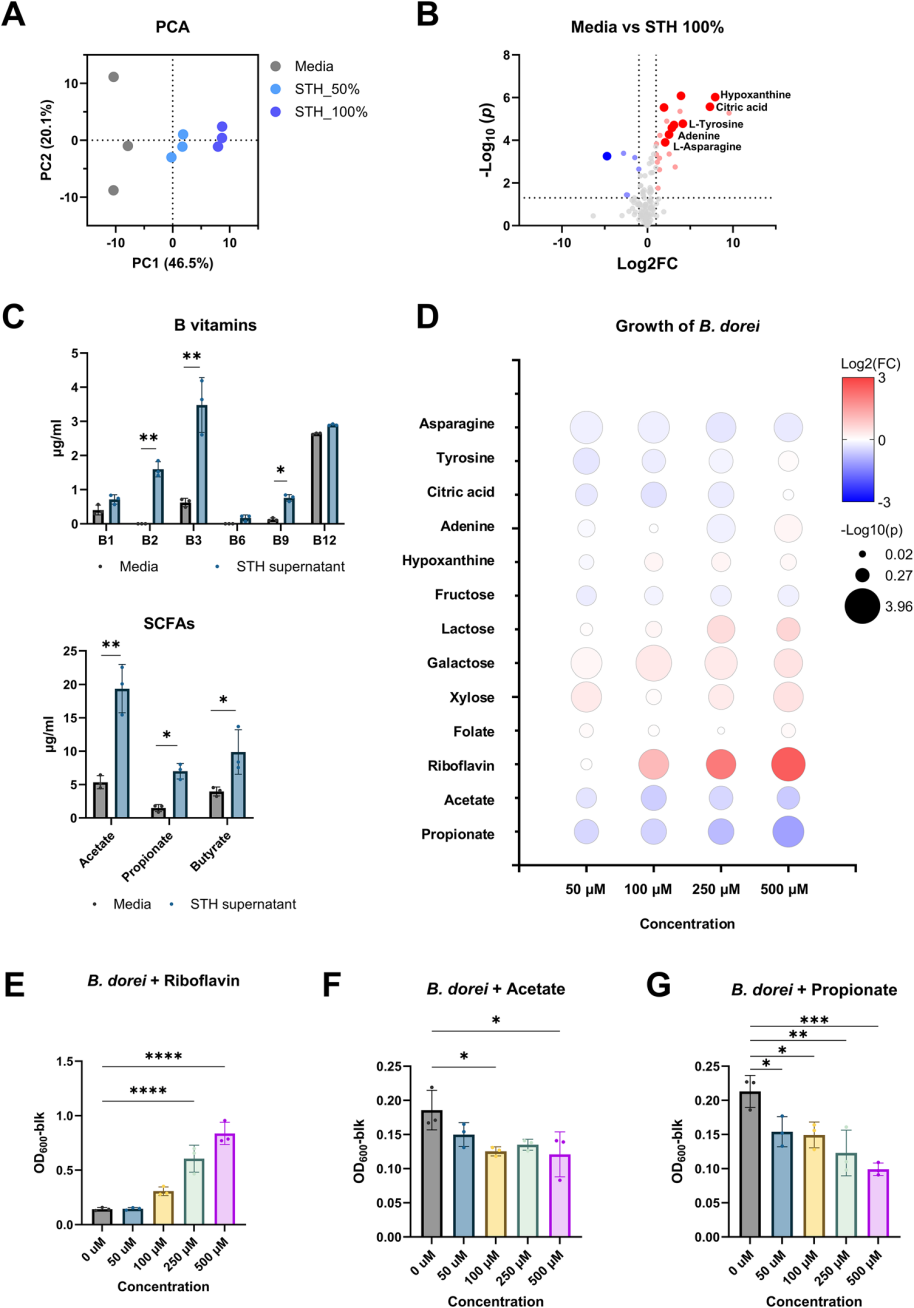
Promotion of *B. dorei* growth by metabolites in the culture supernatant of *S. thermophilus*. (**A**) Analysis of global secretory metabolites in the culture supernatant of *S. thermophilus*. Metabolic profiles were analyzed using PCA scores. (**B**) Volcano plot analysis of differentially abundant metabolites in the culture supernatant of *S. thermophilus* compared to medium. Blue and red dots represent significant decrease and increase in metabolites, respectively (*p* < 0.05). Gray dots represent all other metabolites identified in the dataset of which relative concentrations did not change significantly between the two groups. (**C**) Production of B-vitamins and SCFAs. Significant differences between media and culture supernatant are indicated by asterisks at the 95% (*) and 99% (**) significance levels using Student’s t-test. (**D**) Bubble plot showing fold change of *B. dorei* growth with treatment of selected metabolites compared to control. Bubble color and size indicate fold change and statistical significance using Student’s t-test, respectively. (**E**-G) Growth of *B. dorei* with treatment of riboflavin (**E**), acetate (**F**), and propionate (**G**). Significant differences are indicated by asterisks at the 95% (*), 99% (**), 99.9% (***), and 99.99% (****) significance levels using one-way ANOVA with Dunnett’s post-hoc analysis.

As shown in Fig. 4A, the metabolite profile of the *S. thermophilus* culture supernatant was distinct from that of the medium. To rule out potential technical errors, we also analyzed 50% dilution of the culture supernatant. Twenty-one metabolites were identified as newly synthesized by *S. thermophilus*, and nine metabolites were found at significantly higher concentrations in both the 50% and 100% supernatants compared to the medium (Fig. 4B). Additionally, we used HPLC to quantify SCFAs and B vitamins, which are well-known for their biological functions in the host (Fig. 4C). *S. thermophilus* produced most of these metabolites, with particularly high levels of riboflavin (vitamin B_2_), folate (vitamin B_9_), acetate and propionate.

Among the identified metabolites, we selected nine, including asparagine, tyrosine, citrate, adenine, hypoxanthine, folate, riboflavin, acetate, and propionate, and tested their effects on the growth of *B. dorei* (Fig. 4D). Most metabolites had no effect on *B. dorei* growth, except for riboflavin (Fig. 4E-G). Interestingly, supplementation with acetate and propionate inhibited microbial growth, possibly due to disturbances in anion pools or the uncoupling effects of these organic acids^45,46^. Based on these findings, we conclude that metabolites, specifically riboflavin, in the culture supernatant of *S. thermophilus* may promote the growth of *B. dorei*.

### Co-culture of human gut microbial species with *S. thermophilus* promotes B vitamin secretion

Alterations in microbial community balance lead to changes in gut microbiota-derived metabolites, which in turn influence host metabolism^47^. To simulate the effects of *S. thermophilus* supplementation on the metabolic profiles of human gut microbial species, we analyzed SCFAs and B vitamins, key classes of microbiota-derived metabolites. As depicted in Fig. S5, *S. thermophilus* and *R. intestinalis* produced these metabolites at higher levels than other species. Notably, bacteria from the phylum of Bacillota, such as *E. rectale*, *L. casei*, *Lactobacillus rhamnosus* and *R. intestinalis*, demonstrated superior capabilities as SCFA and B vitamin producers, consistent with previous reports^48^.

**Fig. 5:**
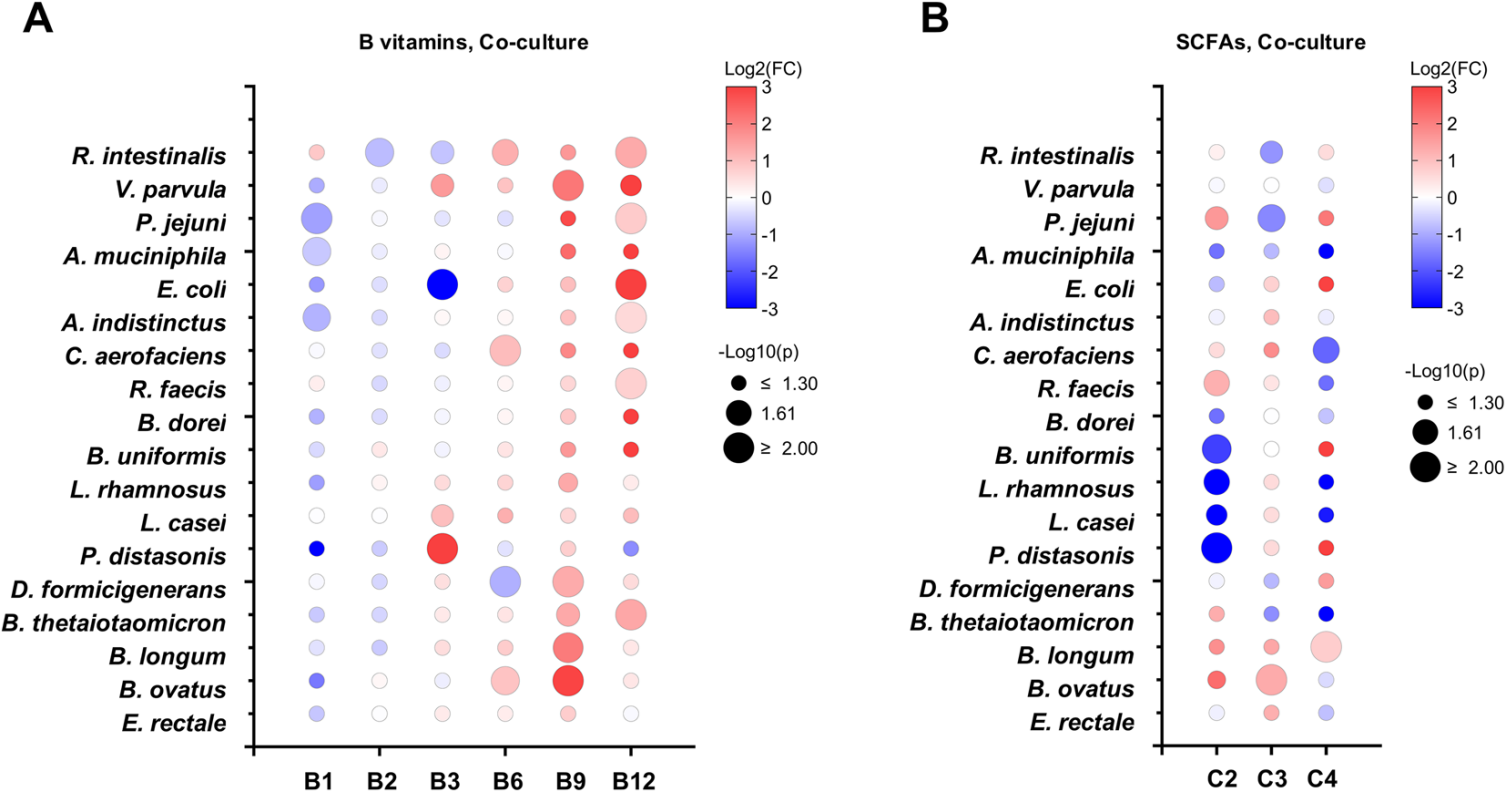
Production of B-vitamins and SCFAs by co-culture of bacterial species composing human gut microbial community with *S. thermophilus*. (A and B) Bubble plot showing fold change of B-vitamins (**A**) and SCFAs (**B**) production in co-culture of bacterial species with *S. thermophilus*. Bubble color and size indicate fold change and statistical significance using Student’s t-test, respectively.

Given the altered growth of several bacteria in co-culture with *S. thermophilus*, we next examined the changes in metabolite concentrations by comparing relative metabolite levels in co-culture versus monoculture (Fig. 5). For *B. dorei*, although its growth benefited from co-culture, no significant changes were observed in its secretory metabolite profile, except for a decrease in acetate. Notably, most bacterial species exhibited significantly enhanced production of pyridoxine and folate, while changes in other metabolites varied by species. In summary, co-culture with *S. thermophilus* influenced B vitamin production, particularly enhancing folate and pyridoxine biosynthesis.

### *S. thermophilus*-derived folate reduces cancer cell viability

**Fig. 6:**
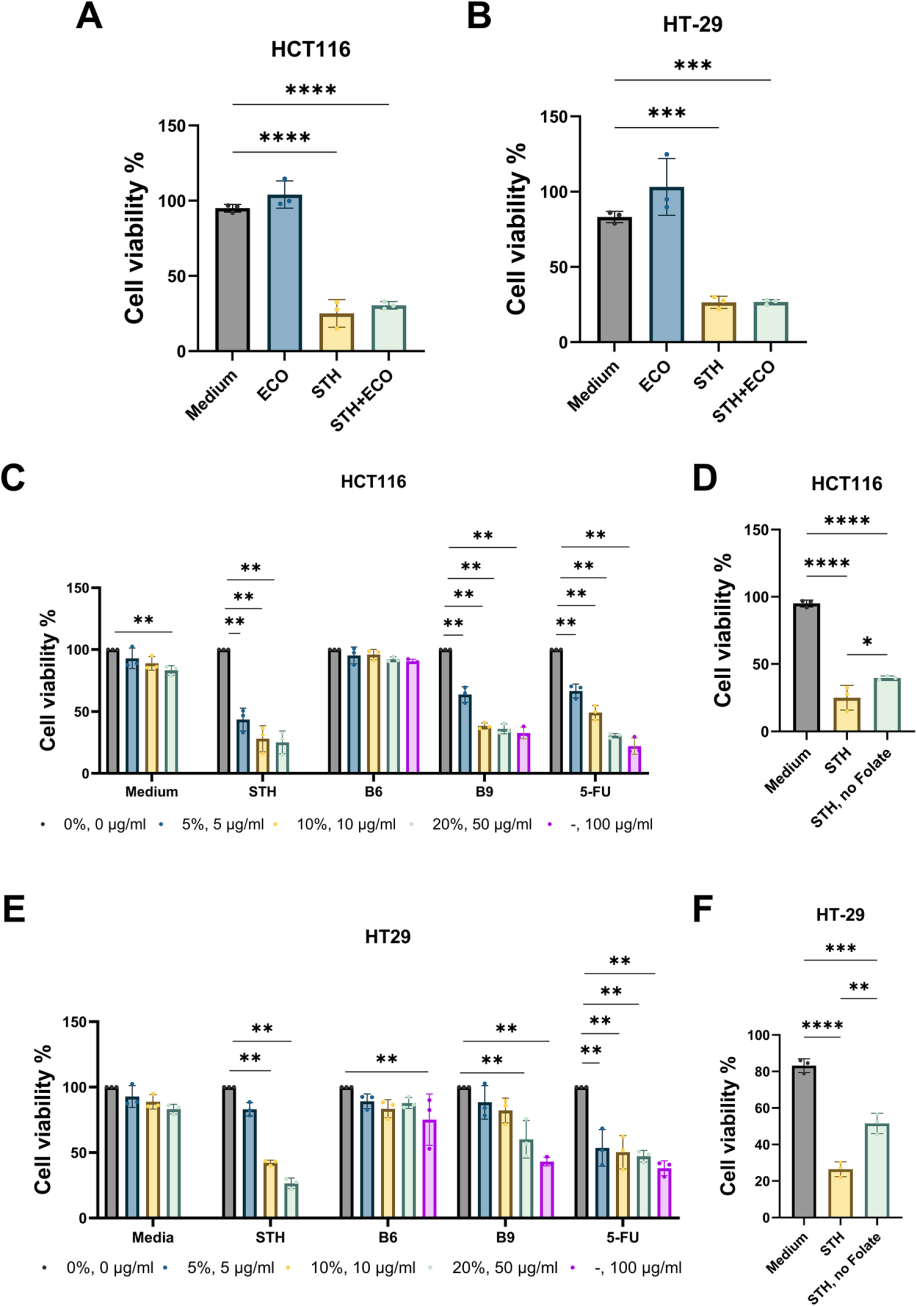
Inhibition of cancer cell viability by *S. thermophilus*-derived folate. (A and B) Viability of cancer cell lines, HCT116 (**A**) and HT-29 (**B**), with the treatment of 20% (v/v) culture supernatant of *E. coli* (ECO) and *S. thermophilus* (STH) compared to medium. (C-F) Viability of cancer cell lines, HCT116 (**C** and D) and HT-29 (E and F), with the treatment of culture supernatant of *S. thermophilus* (STH), pyridoxine (B6), folate (B9), and 5-fluorouracil (5-FU) compared to DPBS control. The culture supernatant of S. thermophilus was prepared with the para-aminobenzoic acid (pABA)-rich and -deficient media (**D** and F). Significant differences are indicated by asterisks at the 95% (*), 99% (**), 99.9% (***), and 99.99% (****) significance levels using one-way ANOVA with Dunnett’s post-hoc analysis.

To investigate the effects of *S. thermophilus* on colon cancer cell viability, we treated HCT116 and HT-29 cells with *S. thermophilus* culture supernatant, using *E. coli* supernatant as a comparison (Fig. 6A and B). Compared to the medium control, *S. thermophilus* strains significantly reduced cell viability, whereas *E. coli* had no effect (Fig. S3B).

Since *S. thermophilus* is a potential source of pyridoxine and folate, we further assessed the effects of these vitamins on cell viability, using 5-fluorouracil (5-FU) as a positive control (Fig. 6C and E). While pyridoxine had no inhibitory effect, folate dose-dependently reduced cancer cell viability, with IC_50_ values of 3.29 µg/ml for HCT116 and 43.73 µg/ml for HT-29.

To further validate the effect of *S. thermophilus*-derived folate on cell viability, we cultured *S. thermophilus* in a para-aminobenzoic acid (pABA)-deficient medium. It is noted that pABA is a precursor for folate biosynthesis, and its absence led to significantly lower folate production by *S. thermophilus*. When colon cancer cells were treated with the supernatant from pABA-deficient *S. thermophilus* cultures, cell viability was significantly higher compared to treatment with normal *S. thermophilus* supernatant (Fig. 6D and F). Taken together, these results suggest that folate produced by *S. thermophilus* reduces cancer cell viability.

## Discussion

Microbial communities are shaped by interactions among individual microbes, ranging from competition to mutualism, with crucial impacts on host health. In the context of human colorectal health, we investigated the interaction between *S. thermophilus* IDCC 2201 and gut commensal bacterial species and found that *S. thermophilus* promoted the growth of *B. dorei*. Several factors derived from *S. thermophilus* were identified, including xylan-hydrolyzing enzymes and B vitamins, which promoted bacterial growth. In addition, the B-vitamin profile produced by gut bacterial species was affected by co-culture with *S. thermophilus*. The bacterial supernatant and specific metabolites reduced the viability of colorectal cancer cells, suggesting that *S. thermophilus* may reorganize the gut microbial community and inhibit colorectal cancer cell proliferation.

*S. thermophilus* has been widely used as a starter culture for dairy products and is one of the most prevalent species in the stool microbiota of westernized populations^38^. In this study, we found that *S. thermophilus* inhibited the growth of most bacterial species in the human gut microbial community when co-cultured. Previous studies have reported that *S. thermophilus* alters microbial composition, for example, by decreasing the abundance of Proteobacteria while increasing specific genera of Firmicutes and Actinobacteria, such as *Lachnoclostridium*, *Lactobacillus*, and *Bifidobacterium*^24,49^. In our co-culture system, we found that *S. thermophilus* decreased the abundance of most bacterial species, while several strains, including *L. casei* and *L. rhamnosus*, remained unaffected. The antimicrobial activity of *S. thermophilus* strains has been reported in several studies^50–53^. In particular, bacteriocins produced by *S. thermophilus*, such as thermopillin 110, have been shown to inhibit the growth of pathogenic bacteria, including *Cutibacterium acnes*, *E. coli*, *Bacillus cereus*, *Pseudomonas fluorescens*, *Staphylococcus aureus*, and *Gardnerella vaginalis*. When we performed a prediction of bacteriocin-encoding genes using the whole-genome sequence of *S. thermophilus* IDCC 2201 (NCBI accession number: CP035306.1) using the BAGEL4 online tool (http://bagel4.molgenrug.nl/)^54^. Three areas of interest were identified in the genome, corresponding to fragments of streptide, BlpU, and sactipeptide. Streptide is a macrocyclic natural product derived from peptides^55^, while BlpU is a broad-spectrum bacteriocin encoded by the bacteriocin-like peptide (blp) gene cluster^56^. Sactipeptide is a type of peptide containing a unique thioether bond, synthesized ribosomally and post-transtionally modified. Recently, streptosactin, a type of sactipeptide, was identified in *Streptococcus* spp., suggesting that *S. thermophilus* may also produce natural antimicrobial compounds that inhibit the growth of specific gut commensal bacteria. In addition to bacteriocins, cell-free supernatant, primarily containing acetic acid, exhibited inhibitory activity against foodborne pathogens. Unlike other bacteria, lactobacilli displayed comparatively higher resistance to *S. thermophilus*. The interaction between lactobacilli and streptococci has been described as complex in several reports. In continuous culture, *Streptococcus mutans* was dominant at pH 7, while *L. casei* predominated at a lower acidic pH^57^. According to Ma et al., *S. thermophilus* suppressed *L. casei* viability, but when *S. thermophilus* was lysed by a phage, *L. casei* exhibited enhanced growth, possibly due to reduced competition for nutrients and the release of intracellular factors from *S. thermophilus*^58^.

Commensalism refers to an interaction where one organism benefits from another without causing harm. In this study, among the bacterial species comprising the human gut commensal community, only *B. dorei* showed positive growth when co-cultured with *S. thermophilus*. *B. dorei*, a member of the *Bacteroides* genus, is known for its roles in inhibiting colonic pathogens, mitigating atherosclerosis, and suppressing inflammation^41^. However, it is noted that despite of beneficial effects of *B. dorei*, such as improving acute colitis and exhibiting antiviral activity, other studies have also suggested its potential pathogenicity, including invasive infectivity and a possible role in promoting type 1 diabetes autoimmunity^41,59^. *Bacteroides* are well known for their ability to utilize polysaccharides, particularly xylan, a β−1,4-linked xylose polymer^60^. When xylose is the sole carbon source, cells require a high amount of metabolic energy to transport and convert it into xylulose, which is subsequently phosphorylated^61^. Due to these energy constraints, most bacterial species exhibit poor xylose catabolism under glucose-limited conditions. However, certain *Bacteroides* strains, including *B. ruminicola* and *B. xylanolyticus* X5-1, have developed adaptations to efficiently utilize xylose, redirecting it into the pentose phosphate pathway in the absence of glucose^61,62^. In the current study, we suggest that *S. thermophilus* may promote the growth of *B. dorei* through several factors, including xylan-hydrolyzing enzymes and B vitamins, although the specific mechanisms remain to be elucidated. A plausible explanation is that xylanase produced by *S. thermophilus* may release xylose as an alternative carbon source, providing a competitive advantage to *B. dorei*, which is well adapted to efficiently utilize xylose compared to another gut microbiota. In the absence of glucose, such as in the physiological environment of the large intestine, *B. dorei* can redirect xylose into the pentose phosphate pathway, supporting the hypothesis of a commensal relationship between *S. thermophilus* and *B. dorei*.

In the current study, we identified *S. thermophilus* as an effective folate producer and an inducer of folate production in gut microbiota. *S. thermophilus* is well known for its folate production, which has been linked to anti-cancer and anti-oxidative activities^25,63^. However, it is also noted that folate intake may have a dual relationship with cancer, acting as a double-edged sword: adequate intake prior to cancer development may have a preventive role, whereas excessive intake after cancer onset may promote carcinogenesis^64,65^. Folate biosynthesis in bacteria involves two metabolic branches that converge to generate dihydropteroate: pABA and DHPPP (6-hydromethyl-7,8-dihydropterin) branches. It has been suggested that dihydroneopterin synthesis, encoded by folQ, may represent a metabolic bottleneck in *S. thermophilus*^63^. Although the catalytic mechanism of folQ is not yet fully understood, it is believed to mediate the removal of pyrophosphate from DHNTP (dihydropterin triphosphate), a step that is often absent in many other bacterial species. In addition, *B. dorei* is a significant contributor to gut folate production^66^. Research indicates that *B. dorei* is strongly associated with folate biosynthesis in infants, which has been linked to enhanced immune function^67^. Given these findings, we hypothesize that *S. thermophilus* shares folate-derived nutrients specifically with *B. dorei*, potentially promoting its growth in healthy individuals. Interestingly, *S. thermophilus* is also known to produce formic acid as a byproduct of glucose fermentation coupled with oxygen consumption^68,69^. Notably, when co-cultured with other lactic acid bacteria such as *Lactobacillus delbrueckii* ssp. *bulgaricus*, formic acid production by *S. thermophilus* increases, facilitating the anaerobic growth of these bacteria and influencing the overall fermentation process. Formic acid also serves as a key intermediate in folate-mediated one-carbon metabolism, where it is directly incorporated into the folate pool via 10-formyl-THF synthase^70,71^. The potential co-production of formic and folic acids by *S. thermophilus* may contribute to one-carbon metabolic functions in both gut microbiota and the host, thereby underscoring the biological importance of *S. thermophilus* in the gut environment.

Furthermore, we confirmed the inhibitory effects of folate and *S. thermophilus* supernatant on the viability of colon cancer cells. Many studies suggest an inverse association between dietary folate intake and CRC risk^72^. Given that folate plays a critical role in DNA synthesis, stability, and integrity, its deficiency may induce DNA strand breaks, impair DNA repair, and increase mutation rates, thereby contributing to the formation of colorectal neoplasms^73^. Recent studies have accumulated evidence showing that *S. thermophilus* inhibits colorectal tumorigenesis^24,74^. However, only a few inhibitory factors have been identified, including the secretion of beta-galactosidase^24^. Here, we propose that folate produced by *S. thermophilus* may be one of the key factors exerting a protective effect against colon cancer.

While our study provides extensive insights into human gut microbe interactions and metabolite profiles, it has certain limitations. First, pairwise co-culture experiments, although useful in elucidating interactions between specific strains, may not fully capture the complexities of higher-order interactions involving multiple species^19^. Second, in vitro models cannot replicate the diverse host factors that influence microbial interactions. Third, while *B. dorei* has recently been reported to have health-promoting functions, it may also exhibit pathogenic potential under specific conditions. Lastly, microbial interactions vary depending on the environmental context within the human host. Considering these limitations, more comprehensive studies investigating the effects of *S. thermophilus* on entire microbial communities are warranted.

In conclusion, we found that *S. thermophilus* affected the growth of most gut commensal bacteria, with exceptions including *B. dorei* and lactobacilli, potentially altering the structure of gut microbial community. The production of beneficial nutrients, such as vitamins and SCFAs, was also modulated by co-culture with *S. thermophilus*. Notably, folate produced by *S. thermophilus* reduced viability of CRC cells. These findings suggest that *S. thermophilus* may reorganize the gut microbial community and inhibit colorectal cancer cell proliferation.

## Supplementary Information

Below is the link to the electronic supplementary material.

Supplementary Material 1

## Data Availability

The data that support the findings of this study are available on request from the corresponding author, MS (mhshin@inha.ac.kr). Raw sequences of NGS data were deposited in the NCBI Sequence Read Archive under project accession number PRJNA1301078.
