# Supplementary material for: The impact of Streptococcus thermophilus IDCC 2201 on gut microbiota and its potential as a prophylactic agent for colorectal cancer
Source: Sci Rep. 2025 Oct 23;15:37140. doi: 10.1038/s41598-025-20976-w (PMC12550040; doi:10.1038/s41598-025-20976-w)

# The impact of *Streptococcus thermophilus* IDCC 2201 on gut microbiota and its potential as a prophylactic agent for colorectal cancer

Eoun Ho Nam[1,2,#], Minjee Lee[3,#], Hayoung Kim[3,#], Donggyu Kim[1,2], Yeji Lee[1,2], Young Hoon Jung[4], Jungwoo Yang[5]*, Minhye Shin[1,2]*

[1]College of Medicine, Inha University, Incheon, 22212, Republic of Korea

[2]Program in Biomedical Science and Engineering, Inha University, Incheon, 22212, Republic of Korea

[3]Ildong Bioscience, Pyeongtaek-si, Gyeonggi-do, 17957, Republic of Korea

[4]Food and Bio-industry Research Institute, School of Food Science & Biotechnology, College of Agriculture and Life Sciences, Kyungpook National University, Daegu 41566, Republic of Korea

[5]Department of Microbiology, College of Medicine, Dongguk University, Gyeongju, 38066, Republic of Korea

[#] Eoun Ho Nam and Minjee Lee contributed equally to this work.

* Corresponding authors: Jungwoo Yang (mqbssjy2@gmail.com), Minhye Shin (mhshin@inha.ac.kr)

# Supplementary information

**Supplemental Table 1.** Bacterial strains used in this study

| Species | Strain | Abbreviation | Origin | Media |
|---|---|---|---|---|
| **Probiotic strains** | | | | |
| *Streptococcus thermophilus* | IDCC 2201 | STH | Ildongbioscience | M-17[e] |
| **Human gut bacterial strains** | | | | |
| *Bifidobacterium longum subsp.infantis* | KCTC 3249 | - | KCTC[i] | BL |
| *Collinsella aerofaciens* | KCTC 15038 | - | KCTC | TSA·heme·$K_3$[f] |
| *Veillonella parvula* | KCTC 5487 | - | KCTC | TSA·heme·$K_3$ |
| *Agathobacter rectale* | KCTC 5835 | - | KCTC | TSA·heme·$K_3$ |
| *Mediterraneibacter faecis* | KCTC 5757 | - | KCTC | TSA·heme·$K_3$ |
| *Dorea formicigenerans* | KCTC 15690 | - | KCTC | TSA·heme·$K_3$ |
| *Lacticaseibacillus rhamnosus* | KCTC 5033 | - | KCTC | MRS |
| *Lacticaseibacillus casei* | KCTC 3109 | - | KCTC | MRS |
| *Roseburia intestinalis* | KCTC 15746 | - | KCTC | TSA·heme·$K_3$ |
| *Bacteroides ovatus* | KCTC 5827 | - | KCTC | CM[g] |
| *Bacteroides dorei* | KCTC 5446 | - | KCTC | TSA·heme·$K_3$ |
| *Bacteroides uniformis* | KCTC 5204 | - | KCTC | TSA·heme·$K_3$ |
| *Prevotella jejuni* | KCTC 15415 | - | KCTC | TSA·heme·$K_3$ |
| *Parabacteroides distasonis* | KCTC 5751 | - | KCTC | CM |
| *Bacteroides thetaiotaomicron* | KCTC 5723 | - | KCTC | TSA·heme·$K_3$ |
| *Akkermansia muciniphila* | KCTC 15667 | - | KCTC | CM |
| *Escherichia coli* | MG 1655 | - | ATCC[j] | LB[h] |

[a]MRS, De Man-Rogosa-Sharpe medium (Difco); [b]BL, medium for maintenance of Bifidobacterium spp. (MB cell); [c]RCM, Reinforced Clostridial medium (MB cell); [d]TSB/TSA, Tryptic soy broth/Tryptic soy agar medium (Difco); [e]M-17, medium for selective growth of Streptococcus spp. (Difco); [f]TSA·heme·$K_3$, Tryptic soy agar with hemin and menadione (MB cell); [g]CM, Chopped meat medium (MB cell); [h]LB, Luria-Bertani medium; [i]KCTC, Korean Collection for Type Cultures; and [j]ATCC, Americal Type Culture Collection

**Supplemental Table 2.** Growth ability of each human gut bacterial strain on various culture media

| Species | MRS | M-17 | LB | CM | TSA | BS | TOS-MUP | BL |
|---|---|---|---|---|---|---|---|---|
| **Probiotic strains** | | | | | | | | |
| *Streptococcus thermophilus* | O | O | X | O | O | O | X | O |
| **Human gut bacterial strains** | | | | | | | | |
| *Bifidobacterium longum subsp.infantis* | O | O | O | O | O | O | O | O |
| *Collinsella aerofaciens* | X | O | O | O | O | X | X | X |
| *Veillonella parvula* | X | O | O | O | O | X | X | X |
| *Agathobacter rectale* | X | O | O | O | O | X | X | X |
| *Mediterraneibacter faecis* | X | O | X | X | O | X | X | X |
| *Dorea formicigenerans* | X | X | X | X | O | X | X | X |
| *Lacticaseibacillus rhamnosus* | O | O | O | O | O | O | O | O |
| *Lacticaseibacillus casei* | O | O | O | O | O | X | X | O |
| *Roseburia intestinalis* | X | X | O | O | O | X | X | X |
| *Bacteroides ovatus* | O | O | O | O | X | O | X | X |
| *Bacteroides dorei* | O | O | X | O | O | X | X | X |
| *Bacteroides uniformis* | X | O | O | O | O | X | X | X |
| *Prevotella jejuni* | X | X | X | O | O | X | X | X |
| *Parabacteroides distasonis* | X | O | O | O | X | X | X | X |
| *Bacteroides thetaiotaomicron* | O | O | O | O | O | O | X | X |
| *Akkermansia muciniphila* | O | O | O | O | X | X | X | X |
| *Escherichia coli* | X | O | O | O | O | X | X | X |

**Supplemental Figure 1**. Construction of the synthetic human gut microbiota. (A) Phylogenetic analysis of the 17 bacterial strains comprising SHGM[17]. (B) Relative abundances of each strain from previously published data derived from Human Microbiome Project. It should be noted that several species representing major bacterial taxa in the community were not included due to the unavailability of corresponding strains in our current collection. (C) Growth profile of each strain in mSHIME media. Abbreviations of each strain are indicated in Table S1.

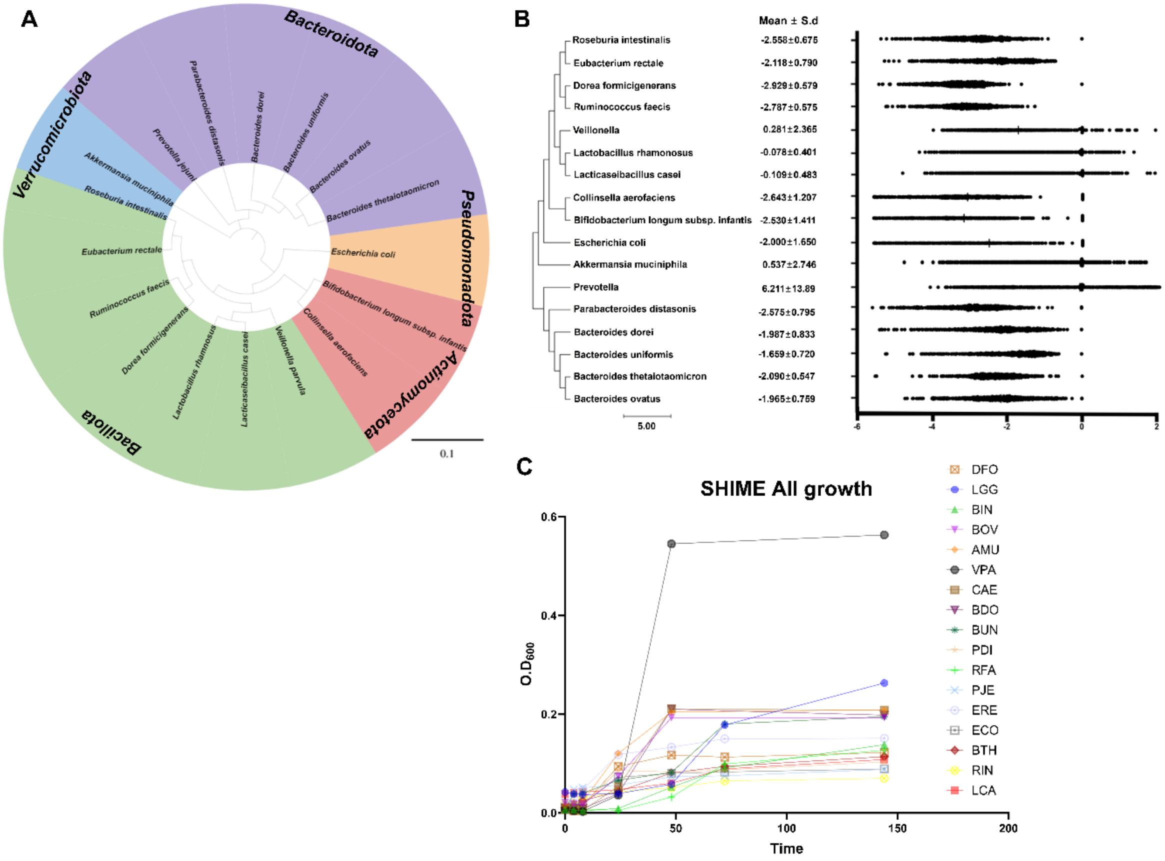

**Supplemental Figure 2**. Interactions among *S. thermophilus* and bacterial strains composing human gut microbial community. Individual abundance ratio ($r_{bm}$) of CFU in co-culture with *S. thermophilus* compared to its mono-culture without *S. thermophilus* was calculated. Significant differences between mono- and co-culture are indicated by asterisks at the 99% (**) significance levels using Student's t-test.

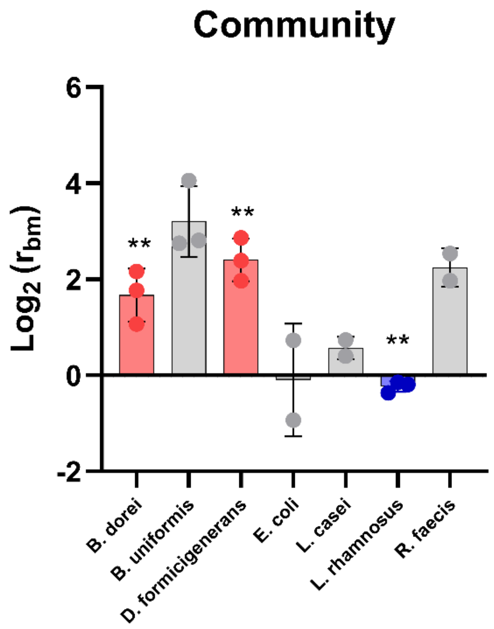

**Supplemental Figure 3**. Effects of *S. thermophilus* strains on *B. dorei* growth and cancer cell viability. (A) Growth of *B. dorei* and (B) viability of cancer cell line, HCT 116, with treatment of the culture supernatant of *S. thermophilus* strains, IDCC 2201, KCTC 21173, and KCTC 3779. Significant differences between control and supernatant treatment are indicated by asterisks at the 95% (*) significance levels using Student's t-test.

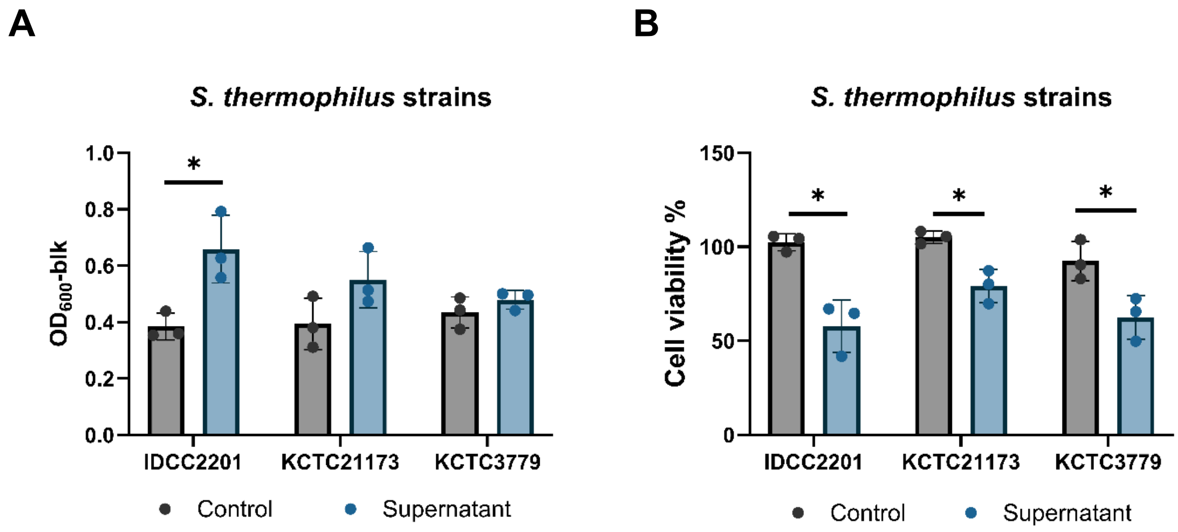

**Supplemental Figure 4**. Heatmap representation of metabolites produced by *S. thermophilus* IDCC 2201. Relative abundance of metabolites in blank medium and *S. thermophilus* culture supernatant was calculated based on unit variance scaling.

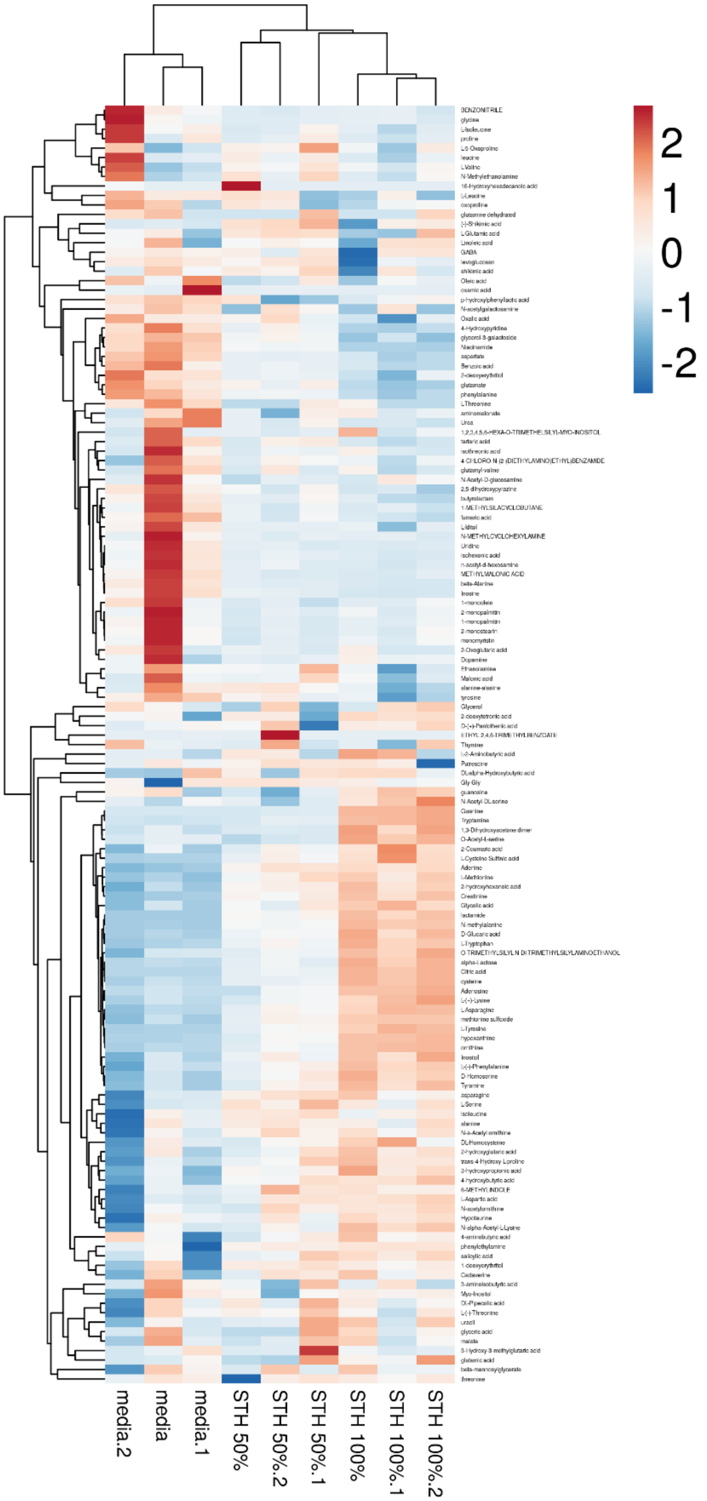

**Supplemental Figure 5**. Heatmap representation of short chain fatty acid and B vitamins production across 17 bacterial strains. Metabolite concentration was normalized based on unit variance scaling. Significant differences between sample and blank medium control were determined using Student's t-test. Abbreviations of each strain are indicated in Table S1.

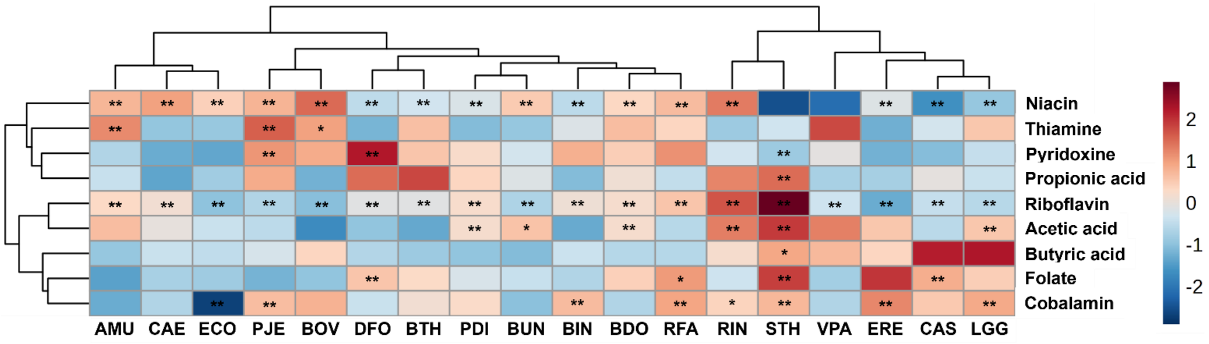

Supplement: Supplementary file 1 — Supplementary Material 1 [file 41598_2025_20976_MOESM1_ESM.pdf]
